# Expressed Emotion in Adolescent Suicidal Ideation: The Mediating Role of Thwarted Belongingness and Perceived Burdensomeness

**DOI:** 10.1111/sltb.70089

**Published:** 2026-03-23

**Authors:** Martina Preisig, Lukasz Smigielski, Isabelle Häberling, Marianne Rizk‐Hildbrand, Tara Semple, Michelle Roth, Lea Hess, Christian Hertel, Michael Kaess, Dagmar Pauli, Susanne Walitza, Gregor Berger

**Affiliations:** ^1^ Child and Adolescent Psychiatry and Psychotherapy Psychiatric University Hospital Zurich Zurich Switzerland; ^2^ Chair for Clinical Psychology for Children/Adolescents and Couples/Families, Department of Psychology University of Zurich Zurich Switzerland; ^3^ University Hospital of Child and Adolescent Psychiatry and Psychotherapy University of Bern Berne Switzerland

**Keywords:** adolescence, expressed emotion, perceived burdensomeness, positivity‐negativity difference, suicidal ideation, thwarted belongingness

## Abstract

**Introduction:**

Suicidal ideation (SI) is a major public health concern among adolescents. Although parental criticism within the *Expressed Emotion* (EE) framework has been linked to SI, the impact of a balance between positive and negative interaction remains understudied. This study introduces the *Positivity‐Negativity Difference of EE* (PND‐EE), investigates its relationship with SI, and examines whether dimensions of the *Interpersonal Theory of Suicide* (IPTS) mediate this relationship.

**Methods:**

The study included a combined clinical and population‐based sample of 46 adolescents (M_age_ = 15.37, SD_age_ = 1.37) and their mothers. Participants completed questionnaires assessing SI and the IPTS dimensions *Thwarted Belongingness* (TB) and *Perceived Burdensomeness* (PB). Mothers completed the *Preschool Five Minute Speech Sample*, which has proven suitable for assessing EE in adolescence. Simple and parallel mediation analyses were conducted using Hayes' PROCESS macro.

**Results:**

Higher PND‐EE was directly associated with lower SI in all analyses. Both TB and PB significantly mediated the effect of PND‐EE on SI in simple mediation analyses, but only PB remained statistically significant in the parallel mediation analysis.

**Conclusions:**

Our findings highlight the potential of PND‐EE as a promising measure of EE in adolescents. Enhancing positive parent‐adolescent interactions and addressing TB and PB in clinical interventions may help reduce adolescent SI.

## 1 Introduction

Suicide is a major public health concern, particularly in adolescents, for whom it is the fourth largest cause of death worldwide (Chaya et al. 2024; WHO World Health Organization 2020). According to a recent meta‐analysis, the global prevalence of suicidal ideation (SI) in adolescents ranges from 14.3% to 22.6%, while the prevalence of suicide attempts lies between 4.6% and 15.8%, depending on the country (Van Meter et al. 2023). A better understanding of risk and protective factors can help identify vulnerable individuals and guide effective prevention and intervention strategies to reduce SI and subsequently prevent suicide attempts and suicide.

Particularly among young individuals, family factors play a crucial role in both protecting against and contributing to SI and behavior (Bodenmann 2016). *Expressed Emotion* (EE; Brown et al. 1958) has been investigated as one specific family risk factor (Rea et al. 2020; Ruscio et al. 2017). EE aims to capture the emotional family climate as reflected in communication styles. Traditionally, EE refers to critical and emotionally over‐involved comments and attitudes toward a family member with a psychiatric diagnosis and is associated with various psychopathologies (Hooley 2007) and increased suicide risk (Demir 2018; Peris and Miklowitz 2015). Criticism reflects disapproval or irritation toward certain behaviors, whereas emotional over‐involvement (EOI) refers to an overly caring, excessively emotional, self‐sacrificing attitude toward the affected individual. Depending on the assessment tool, additional dimensions of EE may be evaluated, including positive dimensions such as warmth (WAR), positive comments (PC), and relationship quality (REL; Ruscio et al. 2017). For more information regarding the EE concept please refer to the Supporting Information.

Since its conceptualization, the EE construct has been broadened to include adolescent samples and outcomes such as SI. EE is also used as a general indicator of family climate, independent of psychiatric diagnoses (Rea et al. 2020; Ruscio et al. 2017). Previous research has shown that EE reliably distinguishes between adults with and without self‐injurious behavior (Hack and Martin 2018; Santos et al. 2009), as well as between children with and without psychiatric diagnoses (Fahrer et al. 2022; Peris and Miklowitz 2015). Importantly, parental criticism and low parental warmth have been linked to SI and behavior in children and adolescents (Connor and Rueter 2006). Conversely, positive parental comments have been associated with reduced psychopathology (McCarty and Weisz 2002). A meta‐analysis by Rea et al. (2020) and a systematic review by Ruscio et al. (2017) indicate that parental criticism is the EE component most strongly associated with SI among adolescents, whereas EOI is less predictive. During adolescence, criticism alone may hold prognostic value similar to that of the broader EE construct (Hagan and Joiner 2017; Hooley 2007).

However, focusing exclusively on criticism may overlook the importance of positive communication. Research on romantic relationships suggests that individual well‐being is influenced more by the balance between positive and negative communication than by criticism alone (Bertoni and Bodenmann 2010; Gottman 1993, 2023). This positivity‐negativity ratio impacts not only the well‐being of adults, but also that of their children (Zemp et al. 2014). It is reasonable to hypothesize that this concept also applies to parent–child relationships. For instance, Lubiewska et al. (2022) found that a balance of maternal positivity and negativity, defined as the difference score between positivity and negativity, was more strongly associated with child insecurity than either positivity or negativity alone. However, no research yet has investigated the association between the positivity‐negativity difference of parental comments, assessed in the context of EE, and adolescent SI.

The *Interpersonal Theory of Suicide* (IPTS; Joiner 2005; Van Orden et al. 2010) offers a useful theoretical framework for understanding the mechanisms contributing to SI and behavior. The IPTS posits that SI arises when two psychological states are present: (1) *Thwarted Belongingness* (TB), characterized by a sense of not being part of a valued social group, such as family or friends, and (2) *Perceived Burdensomeness* (PB), the belief that one is a burden to loved ones, accompanied by the perception that one's death would bring them relief.

Several studies support the IPTS across diverse populations (Chu, Buchman‐Schmitt, et al. 2017; Czyz et al. 2015; Ma et al. 2016; Stewart et al. 2017). A recent systematic review (Kirshenbaum et al. 2024) provides robust evidence that both TB and PB are associated with SI. In addition, another systematic review (Espinosa‐Salido et al. 2021) highlighted that TB and PB often serve as mediators linking various psychological, social, and environmental factors to SI. Hagan and Joiner (2017) applied the IPTS to the context of perceived criticism and SI and behavior. They found that perceived criticism had a significant indirect effect on SI and suicide attempts through its impact on feelings of TB, while PB did not emerge as a significant mediator. The IPTS is particularly relevant in the context of EE and adolescent SI, as it outlines interpersonal processes—TB and PB—that may help explain why family climate is closely related to SI.

This preliminary evidence underscores the need for further research on the relationship between EE dimensions and adolescent SI within the IPTS framework. To ensure adequate representation of SI in the sample of this study, adolescents and their mothers were recruited from both clinical and community populations. Rather than focusing solely on criticism, this study proposes the *Positivity‐Negativity Difference of Expressed‐Emotion* (PND‐EE) as a more nuanced component of EE, capturing the balance between critical and positive family communication. Additionally, to better understand the psychological mechanisms linking family dynamics to SI, this study integrates key constructs of the IPTS (TB and PB). We hypothesized that higher PND‐EE would be directly associated with lower SI, and that TB and PB would mediate the relationship between PND‐EE and SI.

## 2 Materials and Methods

### 2.1 Participants and Procedure

The study sample consisted of 46 adolescents (M_age_ = 15.37, SD_age_ = 1.37, 80.4% female; see Table 1 and Table A for sociodemographic and descriptive characteristics) and their mothers. Recruitment occurred through three channels: (1) adolescents participating in the *Adolescent Attempted Suicide Short Intervention Program* in addition to regular in‐ or outpatient treatment (*n* = 17; AdoASSIP; Berger and Michel 2025; Gysin‐Maillart and Michel 2015), (2) adolescents receiving regular in‐ or outpatient treatment at a large psychiatric clinic in Switzerland (*n* = 7), and (3) adolescents from the general population not currently in treatment (*n* = 22). All participants resided in the greater Zurich area in Switzerland, encompassing both urban and rural settings. Inclusion criteria were as follows: written informed consent from both the adolescent and one legal guardian, sufficient German language skills, and age between 13 and 18 years. Adolescents and their mothers each completed a battery of online questionnaires. Additionally, mothers underwent a 15‐min online interview via the video conferencing service *Zoom* (Version 6.2.11). The study was performed in accordance with the 1964 Helsinki Declaration and its later amendments as well as applicable ethical standards. The local ethics authorities approved all study procedures. This study was embedded in the larger project *AdoASSIP*, which is described in more detail in the Supporting Information.

**TABLE 1:** Sociodemographic and descriptive characteristics of adolescents with and without suicidal ideation.

	Total (*N* = 46)	With suicidal ideation (*n* = 24)	Without suicidal ideation (*n* = 22)	*p*
Age (M (SD))	15.37 (1.37)	15.50 (1.37)	15.27 (1.42)	0.709^a^
Sex (*n* (%))				
Female	37 (80.4%)	18 (75.0%)	19 (86.4%)	0.464^b^
Male	9 (19.6%)	6 (25.0%)	3 (13.6%)	
Current educational status (*n* (%))				
School	35 (76.1%)	17 (70.8%)	18 (81.8%)	1^b^
Apprenticeship	9 (19.6%)	5 (20.8%)	4 (18.2%)	
Unemployed/dropped out	2 (4.4%)	2 (8.4%)	0 (0%)	
Psychiatric‐psychological treatment (*n* (%))				
Yes (AdoASSIP and/or regular)	24 (52.2%)	23 (95.8%)	1 (4.5%)	< 0.001*** ^,^ ^b^
No	22 (47.8%)	1 (4.2%)	21 (95.5%)	
SITBI (M (SD))				
Suicidal ideation (M (SD))	1.36 (1.60)	2.73 (1.16)	0 (0)	< 0.001*** ^,^ ^a^
Suicide attempts (*n* (%))				
Yes	19 (41.3%)	19 (79.2%)	0 (0%)	< 0.001*** ^,^ ^c^
No	27 (58.7%)	5 (20.8%)	22 (100%)	
INQ (M (SD))				
TB (M (SD))	2.86 (1.50)	3.46 (1.33)	2.12 (1.37)	< 0.001*** ^,^ ^a^
PB (M (SD))	2.74 (1.88)	3.67 (1.89)	1.61 (1.17)	< 0.001*** ^,^ ^a^
PFMSS				
Positive comments (M (SD))	5.66 (3.00)	4.23 (2.58)	7.09 (2.72)	< 0.001*** ^,^ ^a^
Negative comments (M (SD))	1.14 (1.30)	1.32 (1.49)	0.95 (1.09)	0.488^a^
PND‐EE (M (SD))	4.52 (3.30)	2.91 (3.18)	6.14 (2.61)	< 0.001*** ^,^ ^a^
Warmth (*n* (%))				
Low/middle	18 (39.1%)	11 (45.8%)	7 (31.8%)	0.331^c^
High	28 (60.9%)	13 (54.2%)	15 (68.2%)	
Relationship quality (*n* (%))				
Low/middle	17 (37%)	13 (54.2%)	4 (18.2%)	0.012* ^,^ ^c^
High	29 (63%)	11 (45.8%)	18 (81.8%)	
EOI (*n* (%))				
Low	38 (82.6%)	18 (75.0%)	20 (90.9%)	0.247^b^
Middle/High	8 (17.4%)	6 (25.0%)	2 (9.1%)	

Abbreviations: EOI, emotional over‐involvement; INQ, interpersonal needs questionnaire; M, mean; PB, perceived burdensomeness; PFMSS, preschool five minute speech sample—expressed emotion; PND‐EE, positivity‐negativity difference of expressed emotion; SD, standard deviation; SITBI; self‐injurious thoughts and behaviors interview; TB, thwarted belongingness.

^a^ Mann Whitney *U* test.

^b^ Fisher's exact test.

^c^ Chi squared test.

* *p* < 0.05.

*** *p* < 0.001.

### 2.2 Measures

#### 2.2.1 Interpersonal Needs Questionnaire (INQ)

The INQ (Van Orden et al. 2012) assesses TB and PB as defined by the IPTS. It is a self‐report measure consisting of 15 items rated on a 7‐point Likert scale. Mean scores were calculated for two subscales (TB and PB), with higher values indicating stronger feelings of TB and PB. We used the German version of the INQ, which has demonstrated good psychometric properties (Glaesmer et al. 2014). The internal consistency in the current sample was excellent (Cronbach's *α* = 0.93 for TB and 0.96 for PB).

#### 2.2.2 Self‐Injurious Thoughts and Behaviors Interview (SITBI)

The SITBI (Nock et al. 2007) measures self‐injurious thoughts and behaviors and is suitable for use with adolescents (Forkmann et al. 2016). In this study, the German version, which has demonstrated good psychometric properties (Fischer et al. 2014), was adapted into a self‐report format. The SITBI consists of six modules, each beginning with a screening question: suicidal thoughts, suicide planning, suicidal gestures, suicide attempts, thoughts of NSSI, and NSSI. The intensity of SI over the past month was assessed using a 5‐point Likert scale, while the presence of suicide attempts was coded as a binary variable (present vs. not present). Due to the questionnaire structure, internal consistency could not be calculated for this instrument. However, previous studies have reported good reliability for the measure (test–retest reliability: Cohen's κ = 0.70 for SI; Nock et al. 2007).

#### 2.2.3 Preschool Five Minute Speech Sample (PFMSS)

This interview measuring observed EE is adapted for the parent–child context and is suitable for use with adolescents (Daley et al. 2003; Scholz et al. 2014). Participating mothers were asked to speak uninterrupted for 5 min about what kind of person their child is and how they get along. Responses were videotaped via *Zoom* (Version 6.2.11) and later coded according to the German translation (Scholz et al. 2014) of Daley's coding manual (Daley et al. 2003). The following categorical EE dimensions were coded: WAR, REL, EOI, each rated as low, middle, or high. Additionally, the number of critical comments (CC) and PC was quantified following the manual's guidelines. This procedure has demonstrated satisfactory inter‐rater reliability in the literature for most dimensions (Cohen's *κ* = 0.73–0.79), except for EOI (Cohen's *κ* = 0.19; Daley et al. 2003).

Furthermore, we used a combined measure of CC and PC, PND‐EE, defined as the number of PC minus the number of CC. This operationalization aligns with Lubiewska et al. (2022), who extended Gottman's original concept beyond the romantic relationship context by applying it to parental positivity and negativity toward a child. We defined PND‐EE as a positive sub‐dimension of EE, with higher PND‐EE values indicating greater positivity relative to criticism in the speech sample. A total EE score was not used, as only two participants (4.35%) met the criteria for high EE. This is consistent with Scholz et al. (2014), who reported that 18% of their clinical sample met the criteria for high EE. Given our combined clinical and community‐based sample, a lower proportion of high EE cases was to be expected. Therefore, we focused on the sub‐dimensions of EE, which show greater variance than the total EE score (see Cartwright et al. 2011; Caspi et al. 2004; Psychogiou et al. 2007 for similar approaches).

All speech samples were transcribed and independently coded by two raters using MAXQDA (VERBI 2022). Inter‐rater reliability was calculated using Intra‐Class‐Correlation (ICC) for metric dimensions and Cohen's κ for categorical dimensions (Wirtz 2004). The calculated inter‐rater reliability was satisfactory to good for CC (ICC = 0.83), PC (ICC = 0.79), and REL (Cohen's *κ* = 0.82), and moderate for WAR (*κ* = 0.46) and EOI (*κ* = 0.57). In cases of discrepancy, the two raters discussed their assessments until consensus was reached.

### 2.3 Statistical Analyses

Analyses were conducted using IBM SPSS Version 30.0 (IBM Corp. 2024), except for power analyses, which were conducted in R (Posit Team 2023). The significance level was set at *p* = 0.05. Prior to statistical testing, missing data were assessed using Little's MCAR test (Little 1988), which indicated that values were missing completely at random (𝜒^2^ = 170.63, df = 175, *p* = 0.579). Accordingly, missing values were imputed using expectation maximization (Dempster et al. 1977). In total, only six values were imputed.

As part of the descriptive analyses, we examined group differences in key variables between adolescents with and without SI. Participants were categorized into two groups based on the SI intensity variable: 0 = no SI (*n* = 22), 1–4 = SI (*n* = 24). For group comparisons, Mann Whitney U tests were computed for continuous variables, as the assumption of normality was not met. For categorical variables, Chi‐squared or Fisher's exact tests were applied, depending on whether the assumption of a minimum expected cell frequency of five was met (Kim 2017). Due to distribution considerations, the categorical EE dimensions—WAR, REL, and EOI—were transformed into binary variables to avoid categories with insufficient frequencies. Specifically, WAR and REL were collapsed into low/middle and high; EOI was collapsed into low and middle/high. This categorization intended to balance group sizes by combining low‐frequency categories, thereby improving group comparability for statistical analysis.

**FIGURE 1:**
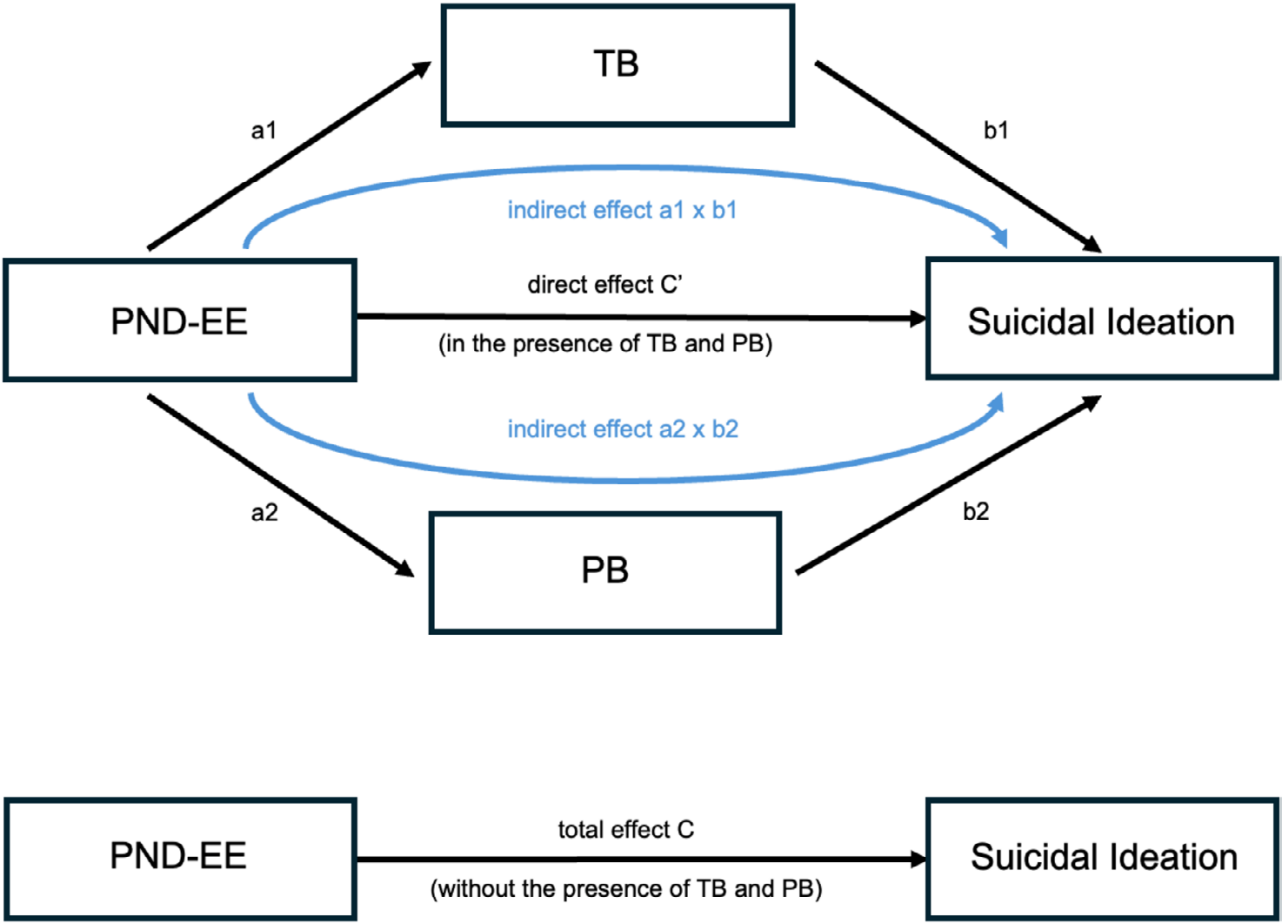
Proposed parallel mediation model depicting the roles of Thwarted Belongingness and Perceived Burdensomeness in the relationship between the Positivity‐Negativity Difference of Expressed Emotion and suicidal ideation. The indirect effect refers to the effect of the independent variable on the dependent variable through the mediator variables. PB, Perceived Burdensomeness; PND‐EE, Positivity‐Negativity Difference of Expressed Emotion; TB, Thwarted Belongingness.

Mediation analyses were employed to test the hypothesis that the effect of PND‐EE on SI is mediated by TB and PB. The analyses were performed using Hayes' PROCESS macro (Hayes 2014), which is based on ordinary least squares regression and is robust to normality violations due to its bootstrapping procedure. We used 10,000 bootstrap iterations and reported 95% percentile bias‐corrected bootstrapped confidence intervals (BCCI) as a robust alternative to *p*‐values. In addition, standardized coefficients and HC3 robust standard errors, as recommended by Davidson and MacKinnon (1993), were reported (Hayes 2014). Age and sex were included as covariates in all models. First, we computed a parallel mediation model, including both TB and PB as mediators, aligning most closely with the IPTS (see Figure 1). Second, we computed two separate simple mediation models for TB and PB, respectively. This approach was chosen due to the small sample size limiting statistical power, and because previous studies suggest that TB and PB may show stronger mediation effects when analyzed independently rather than simultaneously (Espinosa‐Salido et al. 2021). Further, while TB and PB were substantially intercorrelated (Pearson's *r* = 0.83, *p* < 0.001), multicollinearity could be ruled out (see Supporting Information).

As a sensitivity analysis, we conducted a reverse mediation analysis for each of the two simple mediation models, in which the roles of the mediator and the outcome were exchanged (Judd and Sadler 2003; Lemmer and Gollwitzer 2017). This approach aimed to provide additional insights into potential directions of causality. However, it is important to note that no statistical method can determine causal directionality based on cross‐sectional data (Lemmer and Gollwitzer 2017). We did not compare indirect effects between the original and reverse models, as such tests are generally not recommended (Lemmer and Gollwitzer 2017).

In our sample, one participant reporting SI was not receiving treatment, and one participant without SI was receiving treatment. To account for this imbalance, we conducted a further sensitivity analysis in which both participants were excluded and the two simple mediation models (with TB and PB as mediators, respectively) were re‐estimated using the reduced sample (*N* = 44). This approach allowed us to assess the robustness of the findings in more homogeneous groups with fully overlapping SI and treatment status (see Figures C and D in Supporting Information).

Finally, we conducted post hoc power analyses for the total effect of PND‐EE on SI, as well as for the indirect effects in all three mediation models. To our knowledge, no single package allows calculating post hoc power for both total and indirect effects. Therefore, the power for the total effect was computed using the *pwr* package (Champely et al. 2020) in R (Posit Team 2023), while the power for the indirect effects was estimated following the approach by Schoemann et al. (2017), using 10,000 repetitions with 10,000 Monte Carlo draws per repetition. Ideally, a power of 80% (1‐β = 0.80) should be targeted (Field 2009). The results indicated that the total effect of PND‐EE on SI was very well powered (1‐β = 0.99), suggesting a high likelihood of detecting a true effect. In contrast, the power for the indirect effects varied: in the parallel mediation analysis, the indirect effect through TB showed low power (1 − β = 0.20), indicating a high risk of Type II error and suggesting caution in interpreting non‐significant findings. The indirect effect through PB was moderately powered (1 − β = 0.78), implying reasonable confidence in detecting a true effect. The simple mediation analyses showed similar patterns (TB: 1 − β = 0.54; PB: 1 − β = 0.79), indicating that results involving TB may be underpowered and should be interpreted with caution, while findings involving PB are more robust (Field 2009; Serdar et al. 2021).

## 3 Results

### 3.1 Descriptive Characteristics and Group Differences

A correlation matrix of the study variables is included in Table B. Table 1 shows sociodemographic and descriptive characteristics of adolescents with and without SI. Adolescents with SI were significantly more likely to have attempted suicide (*χ* ^2^ = 29.67, *p* < 0.001) and reported significantly higher levels of TB and PB (U = 98, *p* < 0.001; U = 76, p < 0.001, respectively) compared to their peers without SI. Moreover, significant differences were observed for PC (U = 109.5, *p* < 0.001) and PND‐EE (U = 98.5, p < 0.001), with adolescents without SI scoring higher on these dimensions than those with SI. However, even within the SI group, PC exceeded CC. The groups further differed in REL, with mothers of adolescents without SI being more likely to exhibit high REL than mothers of adolescents with SI (*χ* ^2^ = 6.38, *p* = 0.012). No significant differences were observed between the groups for the following variables: CC, WAR, EOI, age, sex, and current educational status.

### 3.2 Parallel Mediation Analysis

**FIGURE 2:**
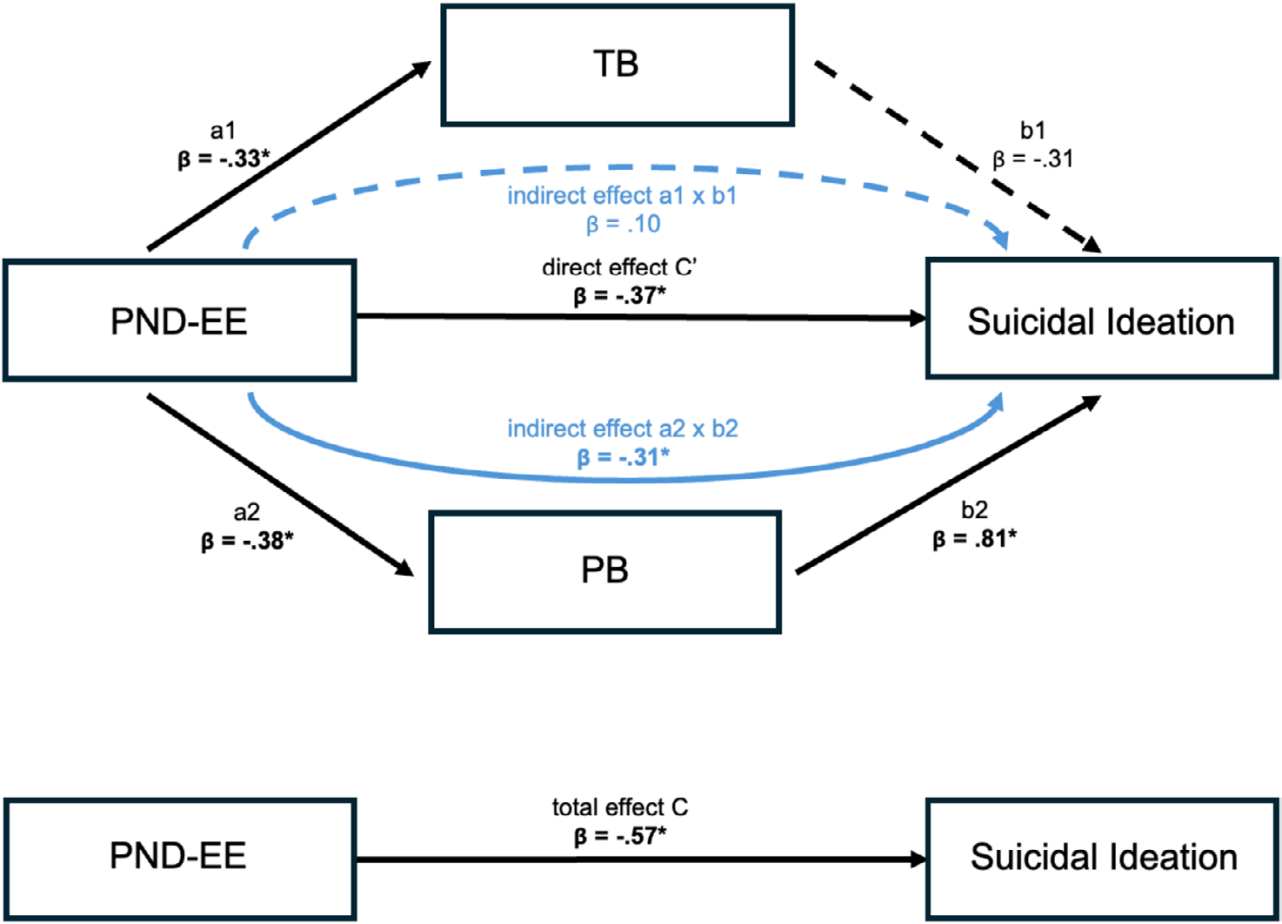
Results of the parallel mediation analysis with Thwarted Belongingness and Perceived Burdensomeness as the parallel mediators between the Positivity‐Negativity Difference of Expressed Emotion and suicidal ideation. Bold lettering and * indicate a significant effect based on 95% percentile bootstrap confidence intervals excluding zero; dashed arrows represent non‐significant paths. β, standardized beta coefficient; PB, Perceived Burdensomeness; PND‐EE, Positivity‐Negativity Difference of Expressed Emotion; TB, Thwarted Belongingness.

The results of the parallel mediation analysis are shown in Figure 2 and Table 2. First, we confirmed a statistically significant total effect of PND‐EE on SI (c: *β* = −0.57, B = −0.28, 95% CI [−0.39, −0.17]), indicating that SI decreases as PND‐EE increases. Importantly, the effect of PND‐EE on SI was mediated by PB (a2xb2: *β* = −0.31, B = −0.15, 95% BCCI [−0.59, −0.09]). Specifically, higher PND‐EE was associated with lower PB (a2: *β* = −0.38, B = −0.22, 95% BCCI [−0.44, −0.06]), which, in turn, was associated with lower SI (b2: *β* = 0.81, B = 0.70, 95% BCCI [0.43, 1.00]). Contrary to our hypothesis, TB did not mediate the effect of PND‐EE on SI (a1xb1: *β* = 0.10, B = 0.05, 95% BCCI [−0.04, 0.30]). However, it should be noted that higher PND‐EE was significantly associated with lower TB (a1: *β* = −0.33, B = −0.15, 95% BCCI [−0.29, −0.04]). Finally, the direct effect of PND‐EE on SI remained significant after accounting for the mediating role of PB and TB (c′: *β* = −0.37, B = −0.18, 95% BCCI [−0.26, −0.07]). The results remained robust when controlling for age and sex.

**TABLE 2:** Path coefficients from the mediation analyses.

	*β*	B	SE	95% CI/BCCI
				LLCI, ULCI
Parallel mediation model TB and PB				
Total effect	−0.57	−0.28	0.05	**−0.39, −0.17**
Direct effect	−0.37	−0.18	0.05	**−0.27, −0.08**
Indirect effects				
TB	0.10	0.05	0.09	−0.04, 0.30
PB	−0.31	−0.15	0.13	**−0.59, −0.09**
Simple mediation model TB				
Total effect	−0.57	−0.28	0.05	**−0.39, −0.17**
Direct effect	−0.46	−0.22	0.05	**−0.33, −0.12**
Indirect effect TB	−0.11	−0.05	0.07	**−0.28, −0.004**
Simple mediation model PB				
Total effect	−0.57	−0.28	0.5	**−0.39, −0.17**
Direct effect	−0.36	−0.17	0.5	**−0.27, −0.08**
Indirect effect PB	−0.21	−0.10	0.09	**−0.42, −0.06**

*Note:* Confidence intervals for indirect and direct effects are 95% percentile bias‐corrected bootstrapped confidence intervals (BCCI). Confidence intervals for total effect are regular 95% confidence intervals (CI).

Abbreviations: B, unstandardized beta coefficient; BBCI, bias‐corrected bootstrapped confidence interval; CI, confidence interval; LLCI, lower limit confidence interval; PB, perceived burdensomeness; SE, standard error; TB, thwarted belongingness; ULCI, upper limit confidence interval; *β*, standardized beta coefficient.

To assess the relative impact of each mediator (TB, PB), we conducted a pairwise contrast analysis of the indirect effects. A statistically significant difference was found (*β* = 0.41, B = 0.20, 95% BCCI [0.09, 0.86]), indicating that PB was a stronger mediator than TB. Additionally, the overall regression model explained 65% of the variance in SI, attributed to PND‐EE, PB, TB, age, and sex (*R* ^2^ = 0.65, *F* = 31.22, *p* < 0.001).

### 3.3 Simple Mediation Analyses

**FIGURE 3:**
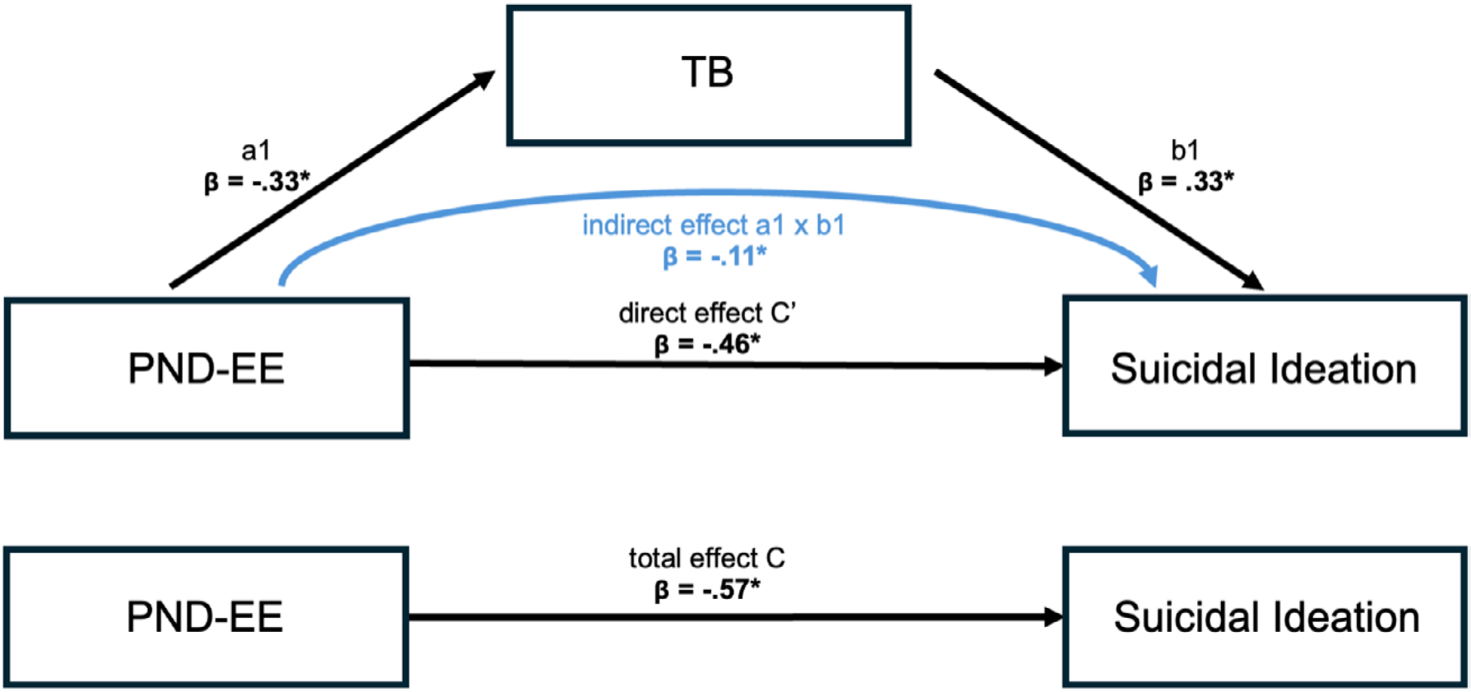
Results of the simple mediation analysis with Thwarted Belongingness as the mediator between the Positivity‐Negativity Difference of Expressed Emotion and suicidal ideation. Bold lettering and * indicate a significant effect based on 95% percentile bootstrap confidence intervals excluding zero. β, standardized beta coefficient; PND‐EE, Positivity‐Negativity Difference of Expressed Emotion; TB, Thwarted Belongingness.

The results of the analysis with TB as the mediator are presented in Figure 3 and Table 2. We first confirmed a significant total effect of PND‐EE on SI (c: *β* = −0.57, B = −0.28, 95% CI [−0.34, −0.13]), indicating that higher PND‐EE was associated with lower SI. The analysis also revealed a significant indirect effect of PND‐EE on SI through TB (axb: *β* = −0.11, B = −0.05, 95% BCCI [−0.15, −0.001]). Specifically, higher PND‐EE was associated with lower TB (a: *β* = −0.33, B = −0.15, 95% BCCI [−0.29, −0.04]), which, in turn, was linked to lower SI (b: *β* = 0.34, B = 0.36, 95% BCCI [0.03, 0.76]). The direct effect of PND‐EE on SI remained significant after accounting for TB as a mediator (c′: *β* = −0.46, B = −0.22, 95% BCCI [−0.33, −0.12]). The covariates age and sex were not statistically significant. The overall regression model explained 46% of the variance in SI, attributed to PND‐EE, TB, age, and sex (*R* ^2^ = 0.46, *F* = 10.25, *p* < 0.001).

**FIGURE 4:**
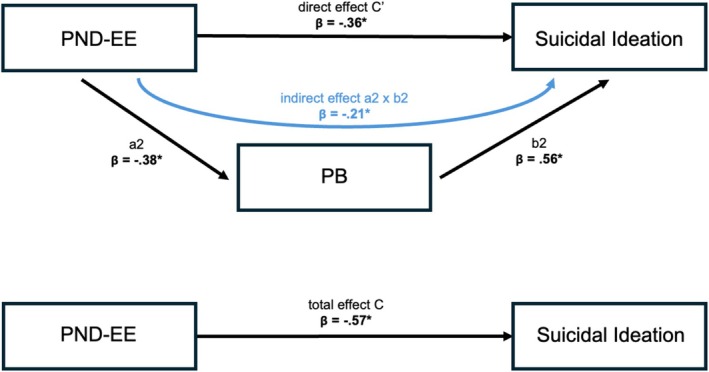
Results of the simple mediation analysis with Perceived Burdensomeness as the mediator between Positivity‐Negativity Difference of Expressed Emotion and suicidal ideation. Bold lettering and * indicate a significant effect based on 95% percentile bootstrap confidence intervals excluding zero. β, standardized beta coefficient; PB, Perceived Burdensomeness; PND‐EE, Positivity‐Negativity Difference of Expressed Emotion.

The results of the analysis with PB as the mediator are presented in Figure 4 and Table 2. The total effect of PND‐EE on SI was statistically significant (c: *β* = −0.57, B = −0.28, 95% CI [−0.39, −0.17]), implying that higher PND‐EE was associated with lower SI. The analysis also revealed a significant indirect effect of PND‐EE on SI through PB (axb: *β* = −0.21, B = −0.10, 95% BCCI [−0.42, −0.06]). Higher PND‐EE was associated with lower PB (a: *β* = −0.38, B = −0.22, 95% BCCI [−0.44, −0.06]), which, in turn, was linked to lower SI (b: *β* = 0.56, B = 0.48, 95% BCCI [0.28, 0.67]). The direct effect of PND‐EE on SI remained significant after accounting for PB (c′: *β* = −0.36, B = −0.17, 95% BCCI [−0.26, −0.07]). The covariates age and sex did not reach statistical significance. The overall regression model explained 62% of the variance in SI, attributed to PND‐EE, PB, age, and sex (*R* ^2^ = 0.62, *F* = 27.62, *p* < 0.001).

The results of the two reverse mediation models with SI as the mediator and TB and PB as the outcome, respectively, are presented in Figures A and B in the Supporting Information. The overall regression model with TB as the outcome explained 28% of the variance in TB, attributed to PND‐EE, SI, age, and sex (*R* ^2^ = 0.28, *F* = 3.99, *p* = 0.008). The overall regression model with PB as the outcome explained 50% of the variance in PB, attributed to PND‐EE, SI, age, and sex (*R* ^2^ = 0.50, *F* = 9.10, *p* < 0.001). We favor the original models not only because of their better model fit, but primarily due to their strong theoretical grounding in the IPTS and the clinical relevance of predicting SI as an outcome. Therefore, the two reverse mediation models are not further discussed.

As a further sensitivity analysis, we re‐ran the two simple mediation models after excluding one participant with SI who was not receiving treatment and one participant without SI who was receiving treatment (*N* = 44). Importantly, the results were highly similar to those obtained in the full sample. The overall regression model with TB as the mediator explained 61% of the variance in SI, attributed to PND‐EE, TB, age, and sex (*R* ^2^ = 0.61, *F* = 15.69, *p* < 0.001). The overall regression model with PB as the mediator explained 73% of the variance in SI, attributed to PND‐EE, PB, age, and sex (*R* ^2^ = 0.73, *F* = 38.42, *p* < 0.001). Given the high similarity of the results, the details of these sensitivity analyses are presented in the Supporting Information (Figures C and D).

## 4 Discussion

This study investigated the relationship between PND‐EE, elements of the IPTS, and SI in a sample of 46 adolescents and their mothers. Mediation analyses showed that higher PND‐EE was directly associated with lower SI and that this relationship was mediated by TB and PB, highlighting their importance as potential targets for interventions aimed at reducing SI among adolescents.

### 4.1 Group Differences Between Adolescents With and Without SI

The levels of PND‐EE and PC were higher in speech samples from mothers of adolescents without SI, and these mothers were more likely to exhibit high REL than those whose adolescents reported SI. Although PC within the EE framework has rarely been studied (Ruscio et al. 2017), our findings align with research linking related constructs, such as parental support, to reduced SI and behavior (Macalli et al. 2018; Miller et al. 2015). Furthermore, higher REL has been associated with less SI and behavior (Kushal et al. 2021) and fewer psychological problems in general (Steele and McKinney 2019).

We found no differences in CC between mothers of adolescents with and without SI. This finding contrasts with a substantial body of research linking perceived criticism to SI (Ruscio et al. 2017), although some studies have reported opposing results (Connor and Rueter 2006; Tarrier et al. 2004). One possible explanation for this result lies in different measurement methods of perceived versus observed criticism—two related but not interchangeable constructs (Miklowitz and Chambless 2015), which are both linked to SI (Ruscio et al. 2017). Adolescents with SI may be more sensitive to criticism and thus perceive higher levels of it compared to their non‐suicidal peers, reflecting the negative cognitive bias frequently observed in psychiatric conditions such as depression (Beck 1979; Everaert et al. 2018).

As shown in Table 1, mothers made a high number of PC (*M* = 5.66, SD = 3.00) and few CC (*M* = 1.14, SD = 1.30). The low frequency of CC may help explain their limited explanatory power. One possible reason for the low amount of CC is selection bias, as particularly supportive parents may have been more likely to participate in the study. A further possible explanation is limited cultural diversity due to the inclusion criterion of German language fluency. Criticism is a cultural construct with its level and style differing significantly between cultures. For example, Western cultures tend to be more direct, whereas Asian cultures tend to be more indirect and polite, reflecting the difference between individualistic and collectivist cultures (Hosseinizadeh and Rassaei Moqadam 2019; Triandis 2018). Nevertheless, the frequencies of CC and PC in our sample are comparable to those reported in the study by Perez et al. (2014), who found an average of M = 2 CC and M = 9.76 PC in a healthy sample. However, the authors reported higher levels of CC (M = 8.9) and lower levels of PC (M = 3.14) in a clinical sample (Perez et al. 2014). The discrepancy in findings may stem from the mixed clinical and community composition of our sample.

Our null finding regarding CC supports the notion that, in the context of adolescent SI, it is not necessarily the expression of CC by parents that plays a pivotal role, but rather the parent's ability to compensate for such comments by maintaining a certain balance of positivity to negativity (PND‐EE). This interpretation aligns with Gottman's (1993, 2023) *Balance Theory*, which posits that relationship stability is not dependent on the amount of negativity, but on the ratio of positivity to negativity (Bodenmann 2016).

Contrary to expectations, WAR did not differ between adolescents with and without SI, which contrasts with previous findings linking WAR to lower internalizing symptoms and SI in adolescents (Connor and Rueter 2006; Manuele et al. 2023). This null finding may be attributed to the low inter‐rater reliability for WAR in our sample as well as the small sample size.

As expected, we did not find any significant group differences for EOI between adolescents with and without SI. This finding is in line with previous research suggesting that EOI is less predictive of SI than criticism, possibly due to its low prevalence and the greater difficulty in detecting this construct in childhood and adolescence (Daley et al. 2003; Rea et al. 2020; Ruscio et al. 2017). Strong emotional involvement and adjustment to daily life in response to an underage child's SI may be adaptive rather than excessive. For instance, Wedig and Nock (2007) found that observed parental criticism, but not EOI, was associated with SI and behavior in adolescents. Moreover, research examining the relationship between EOI and psychiatric symptoms has shown mixed findings. While EOI tends to be more strongly related to internalizing than externalizing symptoms, some studies reviewed by Rea et al. (2020) reported non‐significant associations for both. In some cases, EOI has even been linked to positive outcomes, such as fewer externalizing symptoms (Rea et al. 2020).

### 4.2 Mediating Effect of TB and PB in the Relationship Between PND‐EE and SI

The main finding of this study is that PND‐EE was directly associated with SI, suggesting that higher PND‐EE may be protective against SI, even when accounting for the mediating effects of TB and PB. These findings emphasize the importance of emotional family climate and dyadic parent–adolescent interactions in adolescent SI, consistent with previous research identifying familial problems as the most common antecedent in adolescent suicide cases (Holland et al. 2017). By including not only critical but also positive comments in the PND‐EE construct and thereby expanding the concept of perceived criticism, PND‐EE offers a resource‐oriented perspective on emotional family dynamics. This approach considers not only risk factors but also protective factors, which may be more amenable to change. Similar to research findings in the context of romantic relationships, this balance of emotional expression may help stabilize and improve relationships. When extrapolated to vulnerable adolescents, such a balance may play a significant role in suicide prevention (Bodenmann 2016; Gottman 1993, 2023; Zemp et al. 2014).

In the simple mediation analyses, both TB and PB emerged as significant mediators of the relationship between PND‐EE and SI. These results are in line with a systematic review by Espinosa‐Salido et al. (2021), which identified TB and PB as mediators linking various psychological, social, and environmental factors to SI. For instance, both TB and PB have been found to mediate the effects of insomnia (Chu, Hom, et al. 2017) and perfectionism (Kwan et al. 2017) on SI, while PB has been shown to mediate the association between emotional dysregulation (Rogers and Joiner 2016), interpersonal stress (Buitron et al. 2016), and SI.

In the parallel mediation analysis, only PB emerged as a significant mediator, whereas TB did not. This finding aligns with a review by Kirshenbaum et al. (2024), who identified PB as a stronger predictor of suicidality in clinical samples, and with a review by Ma et al. (2016), who found that PB accounted for more variance in SI than TB. Our findings are also consistent with those of Hirsch et al. (2016), who observed that TB mediated the relationship between chronic pain and suicidal behavior in simple but not in parallel mediation models. On the other hand, our findings contradict those of Hagan and Joiner (2017), who found an indirect effect of perceived criticism on SI and behavior through TB, but not PB. This discrepancy may be due to methodological differences, particularly the use of perceived versus observed measures of EE (Miklowitz and Chambless 2015). Additionally, given the high correlation between TB and PB, the non‐significant TB pathway may reflect reduced statistical power due to shared variance as well as increased model complexity, thus increasing the likelihood of a false negative result (Field 2009).

An important question that has not yet been sufficiently addressed in similar studies (Cole et al. 2013; Hirsch et al. 2016; Puzia et al. 2014) concerns which analytical approach, simple or parallel mediation, is more adequate for research in this field. Theoretically, a parallel mediation model aligns closely with the IPTS and has been frequently applied in previous research (Espinosa‐Salido et al. 2021). However, recent studies suggest that TB and PB may show stronger mediation effects when analyzed independently rather than simultaneously, an observation that challenges the theoretical assumptions of the IPTS and is also discussed by Espinosa‐Salido et al. (2021). Consequently, some studies in the field have employed simple mediation analyses (Cole et al. 2013; Puzia et al. 2014), while others have used and compared both simple and parallel mediation models (Chu et al. 2016; Hirsch et al. 2016), which may represent a sound methodological practice. In our study, two separate simple mediation analyses may be preferred due to the small sample size, limited statistical power, and the high correlation between TB and PB.

### 4.3 Strengths and Limitations

A key strength of this study is the introduction of PND‐EE as a promising construct for understanding and intervening in adolescent SI, especially given its potential for transdiagnostic applicability (Masland and Hooley 2015). In addition, the combination of self‐report and observational measures strengthens our methodology. This is particularly relevant given that the assessment of EE may be susceptible to social desirability effects and observational EE measures are known to be less prone to subjective bias than perceived EE measures (Masland and Hooley 2015; Miklowitz and Chambless 2015). The dyadic design including both adolescents and their mothers further strengthens the methodological rigor of our study.

However, several limitations should be acknowledged. First and foremost, the small sample size limits statistical power, particularly in the parallel mediation analysis. The non‐significant mediation effect through TB should be interpreted with caution, as it may reflect sample size constraints rather than conceptual reasons. Second, the cross‐sectional design precludes causal interpretation. Third, the homogeneity of the sample concerning factors such as age and ethnicity reduced the generalizability of findings. Fourth, only mothers were included in this study, which is why the important role of fathers in adolescents' well‐being could not be taken into account (Islamiah et al. 2023; Temmen and Crockett 2020). Finally, we did not include an overall high versus low EE score in our analyses. However, in the context of our sample, we view the use of subdimensions such as PND‐EE, which display greater variance, as a strength rather than a limitation.

However, PND‐EE itself is subject to certain limitations. Social desirability effects cannot be ruled out. For example, it is possible that parents become less critical after their child's suicide attempt than they were in the time leading up to and potentially influencing the suicide attempt. Furthermore, the construct validity of the newly introduced PND‐EE has not yet been established, and additional research is needed to examine and validate this promising new construct.

### 4.4 Directions for Future Research and Practical Implications

Future research should aim to replicate these findings in larger, longitudinal samples to strengthen causal inferences. It would be valuable to investigate paternal influences, as father‐child relationships may differ from those between mother and child (Bretherton 2010). Additionally, future studies should move beyond SI to examine suicide attempts. Examining low self‐esteem as an additional risk factor and potential mediator in the relationship between PND‐EE and suicide attempts would be of particular interest (Soto‐Sanz et al. 2019). Further, including and comparing both perceived and observed measures of EE could yield deeper insight into parent–child dynamics. Future studies should examine whether potential discrepancies carry clinical relevance, as suggested by prior research (Baumgartner et al. 2020). Additionally, investigating interaction effects between TB and PB, and employing advanced statistical methods such as structural equation modeling, may maximize insights into the dynamic relationships among relevant constructs in this area of research.

In the present sample, only one participant with SI was not receiving treatment, whereas only one participant without SI was receiving treatment. This pattern is not unexpected, as SI frequently co‐occurs with psychiatric disorders such as depression, for which affected individuals are likely to seek treatment (Chaya et al. 2024). Importantly, engagement in psychiatric or psychological treatment may influence communication patterns, and consequently PND‐EE (Girdhar et al. 2024; Lloyd et al. 2023). Future studies with larger samples should explicitly disentangle the effects of SI, treatment status, and PND‐EE by directly comparing adolescents with SI who are receiving treatment to those who are not.

From a clinical perspective, PND‐EE, TB, and PB represent modifiable targets in therapy. Family‐based interventions can be employed to reduce parental criticism and enhance positivity. Psychoeducation plays a crucial role in helping parents understand the potential impact of their attitude and behavior on their children, while specific parenting programs (e.g., Triple P Positive Parenting Program; Sanders et al. 2002) provide opportunities to learn and practice supportive behaviors. Additionally, individual approaches, such as cognitive‐behavioral therapy, may help adolescents reframe their experiences and cope with criticism.

### 4.5 Conclusions

This study investigated the relationship between PND‐EE, components of the IPTS, and SI among adolescents and their mothers. Our findings demonstrate that higher PND‐EE is directly associated with reduced SI and indirectly associated with SI through TB and PB. Group comparisons showed that adolescents with SI had higher levels of TB and PB and were more likely to have previously attempted suicide. Additionally, more PC and higher REL were associated with lower SI, whereas CC and WAR did not significantly differ between groups, possibly due to methodological limitations. Importantly, simple mediation analyses indicated that both TB and PB individually mediated the effect of PND‐EE on SI, while the parallel mediation analysis identified PB as the stronger, independent mediator. Clinically, these findings suggest that improving the ratio of positive to negative parent–adolescent interactions in family interventions, alongside addressing TB and PB in individual therapy, may be effective strategies for reducing adolescent SI and behavior.

## Author Contributions

**Martina Preisig:** conceptualization, investigation, methodology, formal analysis, writing – original draft, visualization. **Lukasz Smigielski:** conceptualization, methodology, validation, writing – review and editing, supervision. **Isabelle Häberling:** conceptualization, investigation, writing – review and editing, supervision, project administration. **Marianne Rizk‐Hildbrand, Tara Semple, Lea Hess, Christian Hertel, Michael Kaess:** investigation, writing – review and editing. **Michelle Roth:** writing – review and editing. **Dagmar Pauli:** investigation, resources, writing – review and editing. **Susanne Walitza:** investigation, resources, funding acquisition, writing – review and editing. **Gregor Berger:** investigation, funding acquisition, supervision, writing – review and editing.

## Funding

This work was supported by Health Promotion Switzerland, PGV03.083.

## Ethics Statement

The study was performed in accordance with the 1964 Helsinki Declaration and its later amendments, as well as applicable ethical standards. All study procedures were approved by the local ethics authorities (Cantonal Ethics Commission—Canton of Zurich, Swissethics number 2022‐01770). All participants and a legal guardian provided informed consent before participation.

## Conflicts of Interest

In the last 5 years, S.W. has received royalties from Thieme, Hogrefe, Kohlhammer, Springer, and Beltz, as well as speaker honoraria from Takeda and Salmon Pharma/MEDICE (since 2023). Her work has been supported by the Swiss National Science Foundation, various EU FP7 programs, HSM (Hochspezialisierte Medizin) of the Canton Zurich (Switzerland), Bfarm Germany, ZInEP, Hartmann Müller Stiftung, Olga Mayenfisch, Gertrud Thalmann, Vontobel, Unicentia, Erika Schwarz, Heuberg Fonds, National Government of Health (BAG), Gesundheitsförderung Schweiz, and Horizon Europe. G.B. has been supported by the Swiss National Science Foundation, the Stanley Foundation, the Gertrud Thalmann Fonds, and the Ebnet Foundation, and has received lecture honoraria from Lundbeck, Opopharma, Antistress AG (Burgerstein) within the last 5 years. Other authors declare no conflicts of interest.

## Supporting information


**Data S1:** sltb70089‐sup‐0001‐Supinfo.docx.

## Data Availability

The data that support the findings of this study are available on request from the corresponding author. The data are not publicly available due to privacy or ethical restrictions.
