# Supplementary material for: Expressed Emotion in Adolescent Suicidal Ideation: The Mediating Role of Thwarted Belongingness and Perceived Burdensomeness
Source: Suicide Life Threat Behav. 2026 Mar 23;56(2):e70089. doi: 10.1111/sltb.70089 (PMC13007485; doi:10.1111/sltb.70089)
Supplement: Supplementary file 1 — Data S1: sltb70089‐sup‐0001‐Supinfo.docx. [file SLTB-56-0-s001.docx]

**Supplementary Information**

**Expressed Emotion (EE)**

EE is a dichotomous construct defined as high versus low EE. EE can be assessed either as subjectively perceived, measured through self-report questionnaires, or as objectively observed, measured through the coding of speech samples (Miklowitz & Chambless, 2015). The first and original measure of observed EE is the *Camberwell Family Interview* (CFI; Brown et al., 1972; Leff & Vaughn, 1985). The CFI is a semi-structured interview that takes approximately one to two hours to administer and is conducted with a patient’s family member in the absence of the patient. The interview is audiotaped and subsequently coded by trained coders. The *Five Minute Speech Sample Expressed Emotion* (FMSS-EE; Magaña et al., 1986) was developed as a time- and cost-effective alternative to the CFI and is the most widely used measure in the field of childhood psychopathology (Sher-Censor, 2015). Studies demonstrate that FMSS-EE scores correspond to CFI scores, although the FMSS-EE tends to underestimate the amount of high EE cases (Magaña et al., 1986). An advantage of the FMSS-EE is that, unlike the CFI, it does not specifically refer to a diagnosis of the person of interest and can therefore also be applied to healthy, non-psychiatric populations (Magaña et al., 1986; Scholz et al., 2014). The *Preschool Five Minute Speech Sample* (PFMSS; Daley et al., 2003; Scholz et al., 2014) is an adaption of the FMSS-EE suitable for preschool-aged children. However, despite its specific name, the PFMSS can also be used with older children and adolescents (Daley et al., 2003; Scholz et al., 2014). The PFMSS is typically administered with a parent of the child or adolescent of interest.

**Temporal stability of EE:** Most studies show modest stability of EE over time, suggesting that EE is neither a stable trait nor merely a representation of a current state. In child and adolescent populations modest but significant stability over time periods of up to two years could be found (Peris & Baker, 2000, Sher-Censor, 2015; Sandberg, Rutter & Järvi, 2003; Miklowitz et al., 2025). For example, Miklowitz and colleagues (2025) found that levels of observed EE were stable in 70% of parents of adolescents (M = 15.7, SD = 1.6) during an 18-month period. EE seems to be moderately stable, however is susceptible to change through circumstances such as symptom severity or intervention efforts (Hooley, 2007; Miklowitz et al., 2025). Therefore, the timing of assessment can have a substantial influence on the level of EE.

**Perceived versus observed measures of EE:** Parent and child reports in the psychiatric and psychological context are often not fully consistent, and the degree of parent - child agreement can itself provide valuable information (Baumgartner et al., 2020). Similarly, regarding the relationship between perceived and observed EE, findings are mixed. Overall, studies show a moderate correlation (Hooley, 2007; Masland & Hooley, 2015; Miklowitz & Chambless, 2015). For example, observed EE correlates moderately with perceived measures of criticism, such as the Perceived Criticism Measure (Hooley, 2007; Masland & Hooley, 2015). However, perceived measures are influenced by subjectivity, individual traits, and previous personal experiences. A more detailed discussion of this topic can be found in the sources mentioned in this paragraph.

**Supplementary Table A**

*Supplementary sociodemographic characteristics of adolescents with and without suicidal ideation*

|  | **Total**  (N = 46) | **With suicidal ideation**  (n = 24) | **Without suicidal ideation**  (n = 22) |
| --- | --- | --- | --- |
| **Gender** (n (%)) |  |  |  |
| *Female* | 36 (78.3%) | 17 (70.8%) | 19 (86.4%) |
| *Male* | 8 (17.4%) | 5 (20.8%) | 3 (13.6%) |
| *Trans female* | 1 (2.2%) | 1 (4.2%) | 0 (0%) |
| *Non-binary* | 1 (2.2%) | 1 (4.2%) | 0 (0%) |
| **Sexual orientation** (n (%)) |  |  |  |
| *Heterosexual* | 35 (76.1%) | 13 (54.2%) | 22 (100%) |
| *Homosexual* | 5 (10.9%) | 5 (20.8%) | 0 (0%) |
| *Bisexual* | 1 (2.2%) | 1 (4.2%) | 0 (0%) |
| *Pansexual* | 1 (2.2%) | 1 (4.2%) | 0 (0%) |
| *Asexual* | 1 (2.2%) | 1 (4.2%) | 0 (0%) |
| *Unsure* | 3 (6.5%) | 3 (12.5%) | 0 (0%) |
| **Ethnicity** (n (%)) |  |  |  |
| *White/Caucasian* | 42 (91.3%) | 21 (87.5%) | 21 (95.5%) |
| *Black* | 1 (2.2%) | 1 (4.2%) | 0 (0%) |
| *Asian* | 1 (2.2%) | 0 (0%) | 1 (4.5%) |
| *Other* | 2 (4.3%) | 2 (8.3%) | 0 (0%) |
| **Monthly family income** (n (%)) |  |  |  |
| *< 5,000 CHF* | 2 (4.3%) | 2 (8.3%) | 0 (0%) |
| *5,000 to 10,000 CHF* | 12 (26.1%) | 6 (25%) | 6 (27.3%) |
| *10,000 to 15,000 CHF* | 16 (34.8%) | 10 (41.7%) | 6 (27.3%) |
| *15,000 to 20,000 CHF* | 6 (13%) | 2 (8.3%) | 4 (18.2%) |
| *> 20,000 CHF* | 4 (8.7%) | 1 (4.2%) | 3 (13.6%) |
| *No information provided* | 6 (13%) | 3 (12.5%) | 3 (13.6%) |
| *Abbreviations:* CHF, Swiss Francs. | | | |

***Adolescent Attempted Suicide Short Intervention Program (AdoASSIP)* Project**

In 2021, the *Adolescent Attempted Suicide Short Intervention Program* (AdoASSIP; Berger & Michel, 2025) was launched, representing the first specialized secondary prevention program in Switzerland specifically targeting adolescents following a suicide attempt. The *AdoASSIP* project integrated both the clinical implementation of *AdoASSIP* and research on risk and protective factors and it is within this project that the current study is embedded. In the clinical context *AdoASSIP* works as an add-on therapy, meaning that all adolescents receiving *AdoASSIP* after a suicide attempt are required to have regular psychiatric-psychological in- or outpatient treatment. For more information on the clinical intervention *AdoASSIP* please see [www.adoassip.uzh.ch](http://www.adoassip.uzh.ch).

Adolescents and their parents who participated in the research assessments of the *AdoASSIP* project received monetary compensation for their participation. Adolescents were given gift vouchers ranging from 10 to 50 Swiss Francs, whereas parents took part in a random draw for a 200 Swiss Franc gift voucher (one voucher was drawn among every 20 participating parents). Participants could choose to complete only the online questionnaires (approximately 2 hours for adolescents and 1 hour for parents) or to additionally take part in face-to-face assessments.

**Online questionnaires:** In addition to demographic questionnaires and the instruments described in the main manuscript, the assessment batteries included well-established and validated questionnaires on topics such as loneliness, trauma, attachment, dyadic coping, conflict, bullying, and clinical symptoms, presented roughly in the listed order. The questionnaires not mentioned in the main manuscript are not relevant to the present study.

**Face-to-face assessments:** For adolescents, this comprised a two-hour in-person session primarily consisting of clinical interviews. Data from these interviews w not used in the current study. For parents, the face-to-face component consisted of a 15-minute online interview conducted via *Zoom* (Version 6.2.11), which included the *Preschool Five Minute Speech Sample* (PFMSS; Daley et al., 2003; Scholz et al., 2014) used in the current study. Additionally, an adapted version of the PFMSS assessing parental support was administered, which is not relevant to the current study. The remaining five minutes of the session were dedicated to technical setup, introductions, and general questions.

**Multicollinearity**Multicollinearity was assessed using the Variance Inflation Factor (VIF) in a linear regression model including PND-EE, TB, PB, age and sex as predictors. According to most authors, VIF is generally preferred over correlations for assessing multicollinearity, with thresholds of <4, <5, or <10 (depending on the source) indicating no problematic multicollinearity (Akinwande et al., 2015; Field, 2009; Hayes, 2014; Midi et al., 2010). Despite the high correlation between TB and PB, all VIF values in our model were within acceptable limits, ranging from 1.01 for age to 3.40 for PB. The correlation matrix of the variables included in the study is presented in Supplementary Table B below.

**Supplementary Table B**

*Correlation matrix of included variables*

|  | **Age** | **Sex** | **Educ.** | **Treat.** | **PND-EE** | **TB** | **PB** | **SI** | **SA** | **PC** | **CC** | **WAR** | **REL** | **EOI** |
| --- | --- | --- | --- | --- | --- | --- | --- | --- | --- | --- | --- | --- | --- | --- |
| **Age** | r=1 |  |  |  |  |  |  |  |  |  |  |  |  |  |
| **Sex** | $r_{\mathrm{pb}}$=.068 | r=1 |  |  |  |  |  |  |  |  |  |  |  |  |
| **Educ.** | ρ=.360* | ρ=.214 | r=1 |  |  |  |  |  |  |  |  |  |  |  |
| **Treat.** | $r_{\mathrm{pb}}$=.036 | φ=.253 | ρ=.146 | r=1 |  |  |  |  |  |  |  |  |  |  |
| **PND-EE** | r=-.049 | $r_{\mathrm{pb}}$=-.078 | ρ=-.127 | $r_{\mathrm{pb}}$=-.514*** | r=1 |  |  |  |  |  |  |  |  |  |
| **TB** | r=.092 | $r_{\mathrm{pb}}$=.182 | ρ=.157 | $r_{\mathrm{pb}}$=.654*** | r=-.343* | r=1 |  |  |  |  |  |  |  |  |
| **PB** | r=.098 | $r_{\mathrm{pb}}$=.069 | ρ=.089 | $r_{\mathrm{pb}}$=.664*** | r=.387** | r=.828** | r=1 |  |  |  |  |  |  |  |
| **SI** | r=.096 | $r_{\mathrm{pb}}$=.175 | ρ=.113 | $r_{\mathrm{pb}}$=.806*** | r=-.584** | r=.512** | r=.704** | r=1 |  |  |  |  |  |  |
| **SA** | $r_{\mathrm{pb}}$=.097 | φ=.031 | ρ=.074 | φ=.803*** | $r_{\mathrm{pb}}$=-.440** | $r_{\mathrm{pb}}$=.387** | $r_{\mathrm{pb}}$=.504*** | $r_{\mathrm{pb}}$=.712*** | r=1 |  |  |  |  |  |
| **PC** | r=.063 | $r_{\mathrm{pb}}$=-.190 | ρ=-.037 | $r_{\mathrm{pb}}$=-.467** | r=.871*** | r=-.254 | r=-.260 | r=-.466** | $r_{\mathrm{pb}}$=-.362* | r=1 |  |  |  |  |
| **CC** | r=.014 | $r_{\mathrm{pb}}$=-.181 | ρ=.052 | $r_{\mathrm{pb}}$=.208 | r=-.472*** | r=.242 | r=.320* | r=.351* | $r_{\mathrm{pb}}$=.246 | r=.023 | r = 1 |  |  |  |
| **WAR** | $r_{\mathrm{pb}}$=-.044 | φ=.059 | ρ=.298* | φ=-.233 | $r_{\mathrm{pb}}$=.357* | $r_{\mathrm{pb}}$=-.176 | $r_{\mathrm{pb}}$=-.076 | $r_{\mathrm{pb}}$=-.144 | φ=-.142 | $r_{\mathrm{pb}}$=.247 | $r_{\mathrm{pb}}$=-.283 | r=1 |  |  |
| **REL** | $r_{\mathrm{pb}}$=.109 | φ=.037 | ρ=.098 | φ=-.372* | $r_{\mathrm{pb}}$=.438** | $r_{\mathrm{pb}}$=-.221 | $r_{\mathrm{pb}}$=-.216 | $r_{\mathrm{pb}}$=-.356* | φ=-.272 | $r_{\mathrm{pb}}$=.317* | $r_{\mathrm{pb}}$=-.321* | φ=.309* | r=1 |  |
| **EOI** | $r_{\mathrm{pb}}$=.044 | φ=.063 | ρ=.000 | φ=.210 | $r_{\mathrm{pb}}$=-.092 | $r_{\mathrm{pb}}$=.233 | $r_{\mathrm{pb}}$=.331* | $r_{\mathrm{pb}}$=.198 | φ=.198 | $r_{\mathrm{pb}}$=-.146 | $r_{\mathrm{pb}}$=-.074 | φ=-.102 | φ=-.005 | r=1 |
| *Note:* r = Pearson correlation coefficient; $r_{\mathrm{pb}}$ = point biserial correlation; φ =Phi coefficient; ρ = Spearman rank correlation; * p < .05, ** p < .01, *** p < .001.  *Abbreviations:* CC, critical comments; Educ., current educational status; EOI, emotional over-involvement; PB, Perceived Burdensomeness; PC, positive comments; PND-EE, Positivity-Negativity Difference of Expressed Emotion; REL, relationship quality; SA, suicide attempts; SI, suicidal ideation; TB, Thwarted Belongingness; Treat., Psychiatric-psychological treatment; WAR, warmth. | | | | | | | | | | | | | | |

**
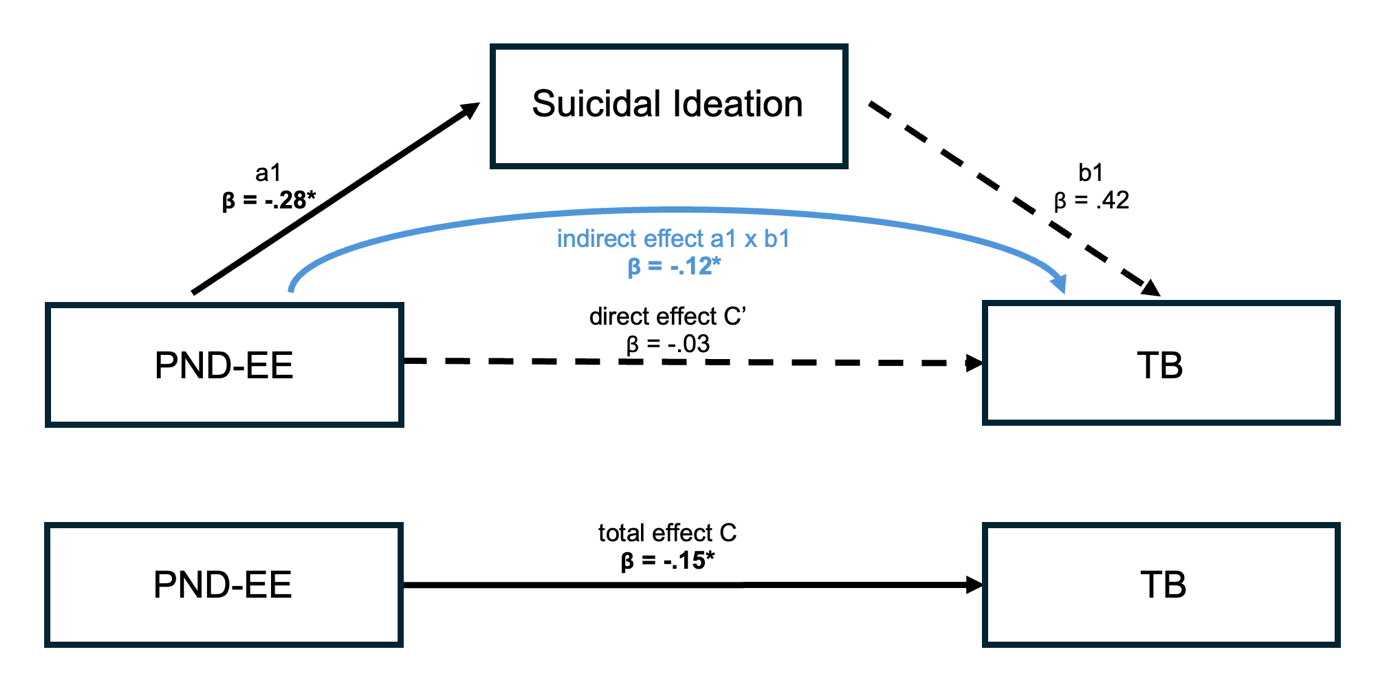
**

*Supplementary Figure A:* Results of the reverse mediation analysis with suicidal ideation as the mediator between the Positivity-Negativity Difference of Expressed Emotion and Thwarted Belongingness.
*Notes:* Bold lettering and * indicate a significant effect based on 95% percentile bootstrap confidence intervals excluding zero; dashed arrows represents non-significant paths.
*Abbreviations:* β, standardized beta coefficient; PND-EE, Positivity-Negativity Difference of Expressed-Emotion; TB, Thwarted Belongingness.

**
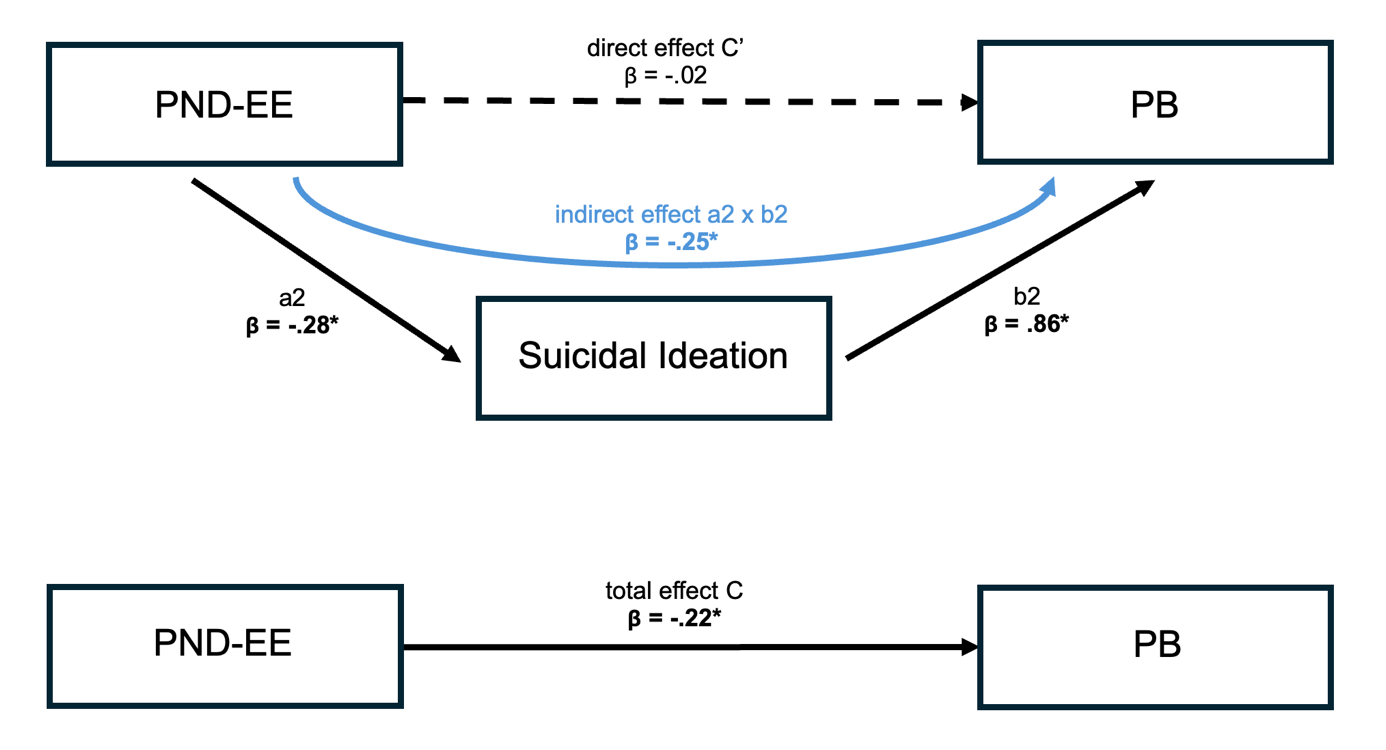
**

*Supplementary Figure B:* Results of the reverse mediation analysis with suicidal ideation as the mediator between the Positivity-Negativity Difference of Expressed Emotion and Perceived Burdensomeness.
*Notes:* Bold lettering and * indicate a significant effect based on 95% percentile bootstrap confidence intervals excluding zero; dashed arrows represents non-significant paths.
*Abbreviations:* β, standardized beta coefficient; PB, Perceived Burdensomeness; PND-EE, Positivity-Negativity Difference of Expressed-Emotion.


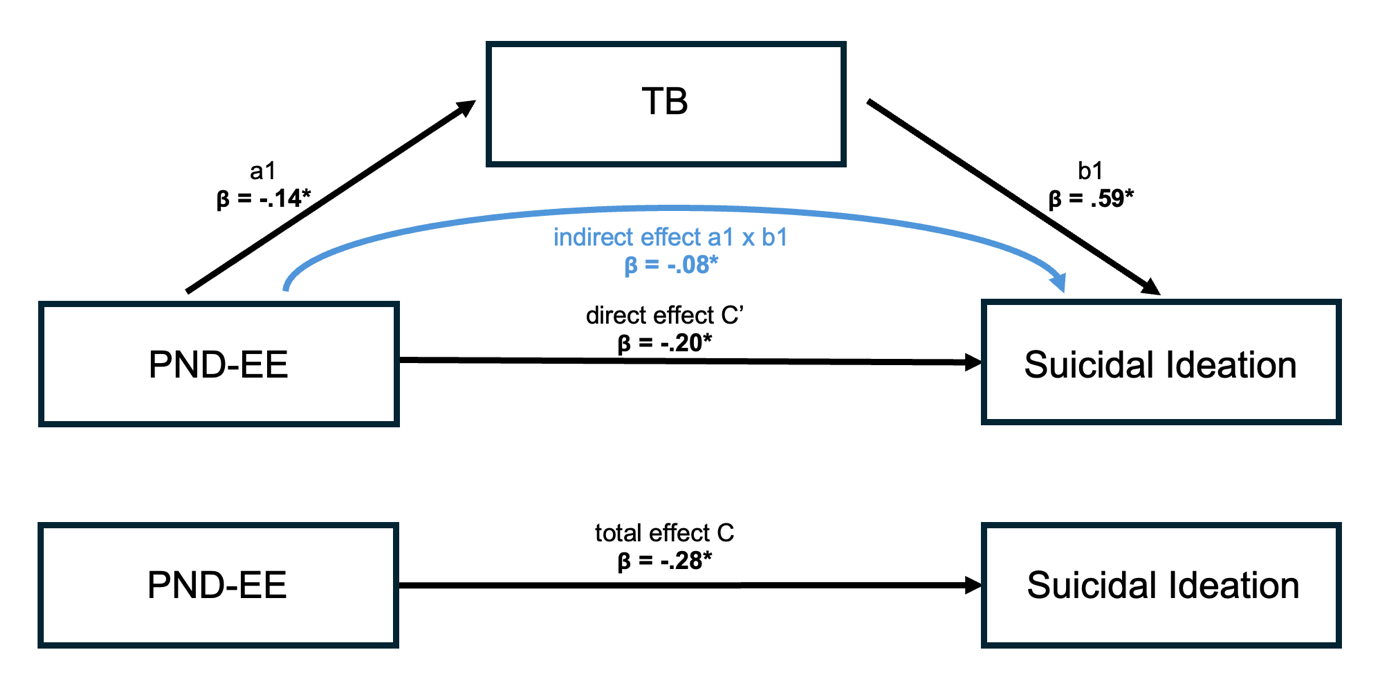


*Supplementary Figure C:* Results of the simple mediation analysis with the reduced sample of N = 44, with Thwarted Belongingness as the mediator between the Positivity-Negativity Difference of Expressed Emotion and suicidal ideation.
*Notes:* Bold lettering and * indicate a significant effect based on 95% percentile bootstrap confidence intervals excluding zero.
*Abbreviations:* β, standardized beta coefficient; TB, Thwarted Belongingness; PND-EE, Positivity-Negativity Difference of Expressed-Emotion.


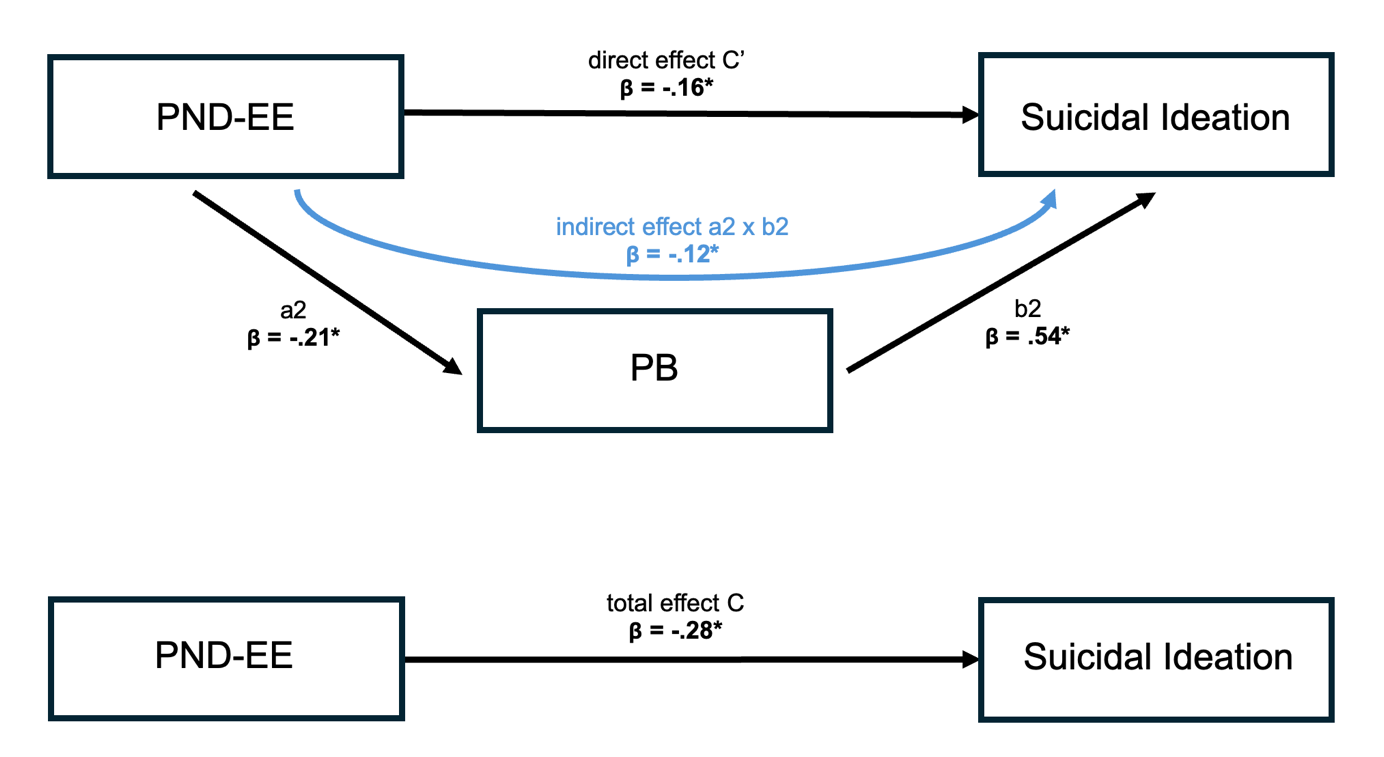


*Supplementary Figure D:* Results of the simple mediation analysis with the reduced sample of N = 44, with Perceived Burdensomeness as the mediator between the Positivity-Negativity Difference of Expressed Emotion and suicidal ideation.
*Notes:* Bold lettering and * indicate a significant effect based on 95% percentile bootstrap confidence intervals excluding zero.
*Abbreviations:* β, standardized beta coefficient; PB, Perceived Burdensomeness; PND-EE, Positivity-Negativity Difference of Expressed-Emotion.

**Supplementary References**

Akinwande, M. O., Dikko, H. G., & Samson, A. (2015). Variance Inflation Factor: As a Condition for the Inclusion of Suppressor Variable(s) in Regression Analysis. *Open Journal of Statistics*, *05*(07), 754–767. <https://doi.org/10.4236/ojs.2015.57075>

Baumgartner, N., Haberling, I., Emery, S., Strumberger, M., Nalani, K., Erb, S., Bachmann, S., Wockel, L., Muller-Knapp, U., Rhiner, B., Contin-Waldvogel, B., Schmeck, K., Walitza, S., & Berger, G. (2020). When parents and children disagree: Informant discrepancies in reports of depressive symptoms in clinical interviews. *J Affect Disord*, *272*, 223–230. https://doi.org/10.1016/j.jad.2020.04.008

Berger, G., & Michel, K. (2025). *AdoASSIP Therapiemanual: Ein Präventionsprogramm für Jugendliche nach einem Suizidversuch*. Unpublished manuscript in publication process.

Brown, G. W., Birley, J. L. T., & Wing, J. K. (1972). Influence of Family Life on the Course of Schizophrenic Disorders: A Replication. *British Journal of Psychiatry*, *121*, 241–258. https://doi.org/10.1192/bjp.121.3.241

Daley, D., Sonuga-Barke, E. J. S., & Thompson, M. (2003). Assessing expressed emotion in mothers of preschool AD/HD children: Psychometric properties of a modified speech sample. *British Journal of Clinical Psychology*, *42*(1), 53–67. https://doi.org/10.1348/014466503762842011

Field, A. (2009). *Discovering Statstics using SPSS* (3rd ed.). Sage Publications.

Hayes, A. F. (2014). *Introduction to mediation, moderation, and conditional process analysis: A regression-based approach.* Guilford Publications.

Hooley, J. M. (2007). Expressed emotion and relapse of psychopathology. *Annual Review of Clinical Psychology*, *3*, 329–352. https://doi.org/10.1146/annurev.clinpsy.2.022305.095236

Leff, J. P., & Vaughn, C. (1985). *Expressed Emotion in Families.* Guilford. https://doi.org/10.1176/appi.psychotherapy.1986.40.4.620

Magaña, A. B., Goldstein, M. J., Karno, M., Miklowitz, D. J., Jenkins, J., & Falloon, I. R. H. (1986). A brief method for assessing expressed emotion in relatives of psychiatric patients. *Psychiatry Research*, *17*(3), 203–212. <https://doi.org/10.1016/0165-1781(86)90049-1>

Masland, S. R., & Hooley, J. M. (2015). Perceived Criticism: A Research Update for Clinical Practitioners. *Clinical Psychology: Science and Practice*, *22*(3), 211–222. https://doi.org/10.1111/cpsp.12110

Midi, H., Sarkar, S. K., & Rana, S. (2010). Collinearity diagnostics of binary logistic regression model. *Journal of Interdisciplinary Mathematics*, *13*(3), 253–267. https://doi.org/10.1080/09720502.2010.10700699

Miklowitz, D. J., & Chambless, D. L. (2015). Perceived Criticism: Biased Patients or Hypercritical Relatives? Commentary on “Perceived Criticism: A Research Update for Clinical Practitioners.” *Clinical Psychology: Science and Practice*, *22*(3), 223–226. https://doi.org/10.1111/CPSP.12108

Miklowitz, D. J., Weintraub, M. J., Denenny, D. M., Merranko, J. A., & Hooley, J. M. (2025). Parental expressed emotion, family conflict, and symptom severity in adolescent offspring of parents with mood disorders. *Journal of Affective Disorders*, 389, 1-10. https:// doi.org/10.1016/j.jad.2025.119620

Peris, T. S., & Baker, B. L. (2000). Applications of the expressed emotion construct to young children with externalizing behavior: Stability and prediction over time. *The Journal of Child Psychology and Psychiatry and Allied Disciplines*, *41*(4), 457-462. https://doi.org/10.1111/1469-7610.00630

Sandberg, S., Rutter, M., & Järvi, J. (2003). Brief measure of expressed emotion: Internal consistency and stability over time. *International Journal of Methods in Psychiatric Research*, 12(4), 182-191. https://doi.org/10.1002/mpr.155

Scholz, K. K., Schuh, L. C., & Döpfner, M. (2014). Manual zur deutschsprachigen Fassung Five Minute Speech Sample für das Kindesalter nach Daley (FMSS-K). *Klinik Für Psychiatrie Und Psychotherapie Des Kindes- Und Jugendalters an Der Universitätsklinik Köln.* Retrieved 2025, October, from https://d-nb.info/1078159718/34

Sher-Censor, E. (2015). Five Minute Speech Sample in developmental research: A review. *Developmental Review*, *36*, 127–155. https://doi.org/10.1016/j.dr.2015.01.005
